# Local Scale Biogeographic Variation in the Magnolia (*Magnolia grandiflora*) Phyllosphere

**DOI:** 10.3390/microorganisms12122546

**Published:** 2024-12-11

**Authors:** Stephanie N. Vaughn, Elizabeth M. Eckard, Venkat K. Kota, Kurre T. Luber, Colin R. Jackson

**Affiliations:** Department of Biology, University of Mississippi, University, MS 38677, USA; snvaughn@olemiss.edu (S.N.V.);

**Keywords:** phyllosphere, microbiome, biogeography, *Magnolia grandiflora*, 16S rRNA

## Abstract

The phyllosphere (aerial plant surfaces colonized by microorganisms) remains an understudied ecosystem in terms of bacterial biogeography, particularly at intermediate or local spatial scales. This study characterized the phyllosphere bacterial community on the leaves of 87 *Magnolia grandiflora* trees sampled throughout a small town, encompassing an area of approximately 60 km^2^. Sequencing of the 16S ribosomal RNA gene revealed the dominant bacterial phyla to be Alphaproteobacteria, Bacteroidetes, and Acidobacteria, consistent with other studies of the phyllosphere. There was a small but significant relationship between the phyllosphere community similarity and the distance between the trees (i.e., trees further apart were more likely to have dissimilar bacterial communities). There was also a relationship between the assigned categories of tree height (low, medium, high) and the phyllosphere bacterial community composition, with the trees in the high category having more diverse bacterial communities on their leaves than the shorter trees. This study provides insight into the relationship between phyllosphere community composition and host tree characteristics and shows that the distance between *M. grandiflora* trees has a significant, albeit low, influence on bacterial composition. These findings contribute to a deeper understanding of phyllosphere microbiome biogeography, highlighting how individual tree characteristics and spatial proximity shape phyllosphere bacterial communities.

## 1. Introduction

Every plant hosts a complex community of microorganisms, collectively referred to as the plant microbiome [1]. This intricate network of microorganisms plays important roles in the health and development of the host plant [2]. The plant microbiome is typically considered to consist of two major components: the rhizosphere, or the microbial community residing on and around the plant’s roots, and the phyllosphere, the microbial community inhabiting aboveground surfaces, primarily leaves [1,3,4]. These distinct habitats offer different environments for microbial growth due to differing moisture, light, and nutrient availability, which place different selective pressures on the microorganisms that inhabit them [4,5]. While much of the early research on plant microbiomes focuses on the rhizosphere, particularly in an agricultural context, there is a growing recognition of the importance of the phyllosphere as a critical habitat for plant-associated microorganisms [2,6,7,8,9].

The phyllosphere consists collectively of a vast surface area of leaves, stems, flowers, and fruits that serves as one of the largest environments for microbial life [3,10], potentially accounting for 60% of the Earth’s total biomass [3,11,12]. Many species of bacteria, Archaea, and microbial Eukarya are adapted for life in the phyllosphere, despite it being an environment subject to fluctuations in environmental conditions such as moisture [7,13], UV radiation [5,14] and temperature [3,15]. These changing conditions influence the microbial communities residing in the phyllosphere [4,7,16]. Interactions between the host plant and their phyllosphere microbiome are also important, and phyllosphere bacterial communities can be shaped by biotic factors such as host plant species, genetics, age, and chemical composition [1,4,17].

While the characteristics of the host plant and environmental conditions at the leaf surface undoubtedly influence the composition of the phyllosphere microbiome, at a broader ecological level, the location of a plant and its proximity to other plants of the same species are also likely to influence the phyllosphere microbial community [1,18,19]. Such spatial patterns are part of the broader field of microbial biogeography [20,21,22], and plant-associated microbial communities have been found to show biogeographic patterns over a variety of scales [1,23,24]. Even when considering factors such as plant species and climate, the geographical distance between hosts is an important factor influencing phyllosphere microbial community composition [18,24,25,26].

In a previous study [26], our research group examined the biogeographic patterns in the phyllosphere communities on *Magnolia grandiflora* L. (Southern Magnolia) trees in a small forest. That study showed that spatial variation over a small area (0.2 km^2^) in a woodland ecosystem influenced the beta diversity of the magnolia phyllosphere, such that the bacterial communities on the trees that were closer together exhibited a higher similarity in bacterial community structure [26]. Other biogeographic studies of the phyllosphere have tended to examine microbial community patterns at much larger spatial scales [24,25,27,28] but report similar trends of plants that are closer together hosting more similar bacterial communities. However, studies at larger biogeographic scales are potentially confounded by a variation in climactic and other environmental factors, and there is a gap in our understanding of the intermediate-scale geographic patterns in phyllosphere communities [23]. In this study, we examined the biogeographic patterns in phyllosphere communities over local/intermediate scales, determining the effect of distance on the phyllosphere bacterial community of a single tree species (*M. grandiflora*) over an area of approximately 60 km^2^. Sampling over such a spatial scale, on a single day, should allow for the determination of biogeographic patterns under more uniform climactic conditions, and allow for greater emphasis in determining the role of spatial patterns and/or local environmental heterogeneity on the phyllosphere community. We hypothesized that the distance between the trees would exert the greatest influence on the composition of the magnolia phyllosphere, leading to a higher dissimilarity on the bacterial phyllosphere composition between the trees that were further apart.

## 2. Materials and Methods

**Figure 1:**
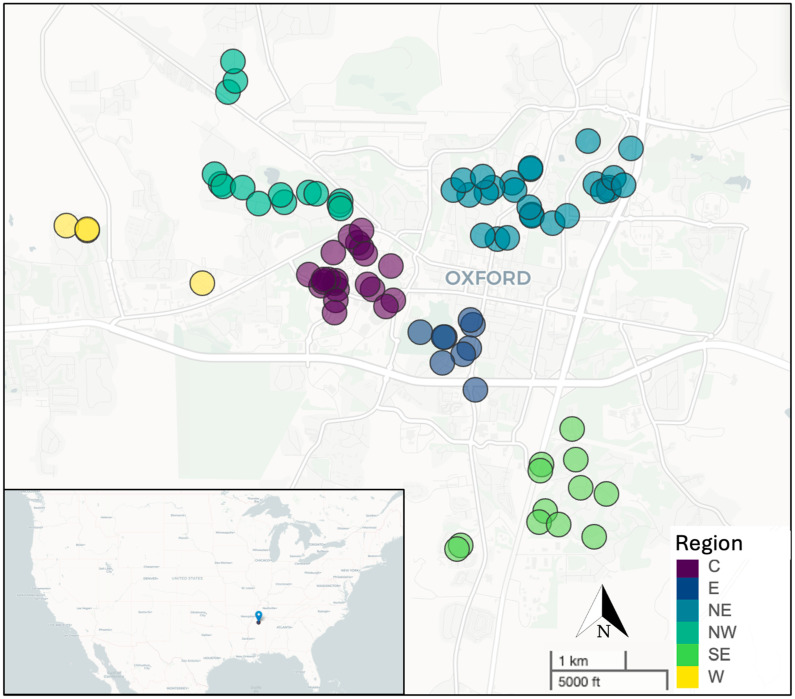
Map of the study area in Oxford, Mississippi, USA, showing the locations of 87 mature southern magnolia (*Magnolia grandiflora*) trees whose bacterial phyllosphere community was characterized through 16S rRNA gene sequencing. Trees were sampled from six different regions within an approximately 60 km^2^ area. Regions were assigned based on their direction relative to the trees designated as the center (C) region, with the surrounding areas categorized as east (E), northeast (NE), northwest (NW), southeast (SE), and west (W).

Leaves were collected from 98 *Magnolia grandiflora* trees throughout the city of Oxford, MS, USA, with 87 trees being used in the final analysis (see below). Oxford is a small city (population approximately 25,000) in the southeastern United States with a humid, subtropical climate of warm summers and mild winters. This serves as a suitable climate for *M. grandiflora*, although it is towards the northern end of the natural range [29]. To minimize variation due to temporal effects, all the trees were sampled on a single day: 5 March 2023. There was no precipitation over the five days prior to sampling or during sampling, and the air temperature over the time of sampling ranged from 16.7 to 24.0 °C. The locations of trees sampled were defined as six regions across the city (Figure 1). The regions were referred to as the center (containing 21 trees; centered on GPS coordinates 34.36662, −89.5383), east (9 trees; 34.3582, −89.5233), northeast (26 trees; 34.37607, −89.5117), northwest (15 trees; 34.37865, −89.5492), southeast (12 trees; 34.34066, −89.5104), and west (4 trees; 34.37045, −89.5717). Specific trees were chosen based on their accessibility and to represent a distribution across most of the study area. While the focus of our study was on the effect of the distance between the trees on the phyllosphere community, we also made brief categorical estimates of the tree properties (height, width) and location characteristics (the amount of shade from other trees and/or buildings, the degree of development from roads and buildings, distance to a road). To allow for all 98 trees to be sampled on a single day, these estimates were limited to assigning categorical scores of 1 (low) to 3 (high) for the estimated height, width, amount of shade, and development. The distance to a road was measured for the trees assigned categorical scores of 1 or 2 for development, but not for those scored with a 3 (no road visible) for that category. A photograph of the tree was taken, and the GPS coordinates were recorded.

Three leaves were collected from each of the trees, with each leaf being collected from a branch 1.5–1.8 m above the ground, and the three leaves being evenly spaced around the tree. Sampling occurred while wearing latex gloves, using sterilized (95% ethanol) scissors, and the three leaves from each tree were immediately placed together in a sterile sample bag. The leaves were transported to the laboratory (roughly the center of the study area) and frozen at −20 °C prior to subsequent processing.

### 2.1. DNA Extraction, Amplification, and Sequencing

DNA was extracted from the phyllosphere following the procedures of Stone and Jackson [26]. Briefly, the leaves were brushed for 1 min with a sterile toothbrush into 6 mL of sterile TE (pH 8.0) buffer, with the three leaves from each tree being combined. The resulting suspensions of the cells were then centrifuged (7000× *g*, 2 min) and the supernatant discarded. The cell pellets were frozen until DNA extraction. DNA was extracted using a PowerSoil Pro DNA extraction kit (Qiagen, Germantown, MD, USA) and the presence of DNA was confirmed by agarose gel electrophoresis.

A dual-index barcoding approach was used for Illumina next-generation sequencing [30]. The procedures generally followed those described by Jackson et al. [31]. Briefly, each sample was amplified with bacterial specific 16S rRNA gene primers that amplified a 250-nucleotide long region of the V4 variable region of the 16S rRNA gene [30]. Each primer was tagged with a specific 8-nucleotide barcode to allow the subsequent pooling of the samples. Following amplification, the barcoded amplicons were standardized using SequalPrep plates (Life Technologies, Grand Island, NY, USA), and pooled prior to sequencing. The assembled library was spiked with 20% PhiX [31] and sequenced using an Illumina NextSeq (Illumina, San Diego, CA, USA) at the University of Mississippi Medical Center’s (UMMC) Molecular and Genomics Core Facility.

Raw sequence files (fastq) were downloaded and processed using the standard 16S rRNA pipeline of the DADA2 package version 1.26.0 within R version 4.2.2 [32]. The sequences were trimmed at truncLen = c(240,180) basepairs (bp) and the quality profile plots were inspected to ensure proper quality of the trimmed reads. Forward and reverse reads were merged with minLen = 135 bp, and the sequences shorter than 250 bp or longer than 256 bp were removed, as were the sequences identified as potential chimeras, chloroplasts, mitochondria, Archaea, or Eukarya. The sequences were classified against the Ribosomal Database Project (RDP) v.18 database (release date July 2020) [33] and the individual sequence types were identified as amplicon sequence variants (ASVs). After clustering the sequences into ASVs, 11 trees were removed from the downstream analyses because of low sequence counts. The samples from the remaining 87 trees were rarefied to 5000 sequences for community analyses.

### 2.2. Statistical Analyses

All the statistical analyses were performed in R version 4.2.2. A map of the sampling sites and regions of each tree was created using the *mapview* function from the “mapview” package [34]. Bacterial sequence data recovered from the *M. grandiflora* phyllosphere were rarefied using the *Rarefy* function of the “GUniFrac” package [35]. Good’s coverage score was estimated to determine the mean bacterial sequencing depth of each sample using the *phyloseq_coverage* function of the “metagMisc” package [36]. The bacterial species richness (species observed), bacterial species diversity (Inverse Simpson’s Index), and bacterial species evenness (Shannon’s Index) were calculated using the *estimate_richness* function of the “phyloseq” package [37]. Analysis of variance (ANOVA) tests were performed to assess the differences in the alpha diversity metrics based on the tree region, and were assigned the categories of tree height, width, shade, and development. For the trees in which the distance to a road was measured, the relationship between that distance and the alpha diversity metrics was analyzed using Pearson’s correlation. The relative abundance of the major bacterial taxa (phyla or families ≥1.0% relative abundance of all the sequences) was determined and analyzed for the differences in their proportions in the phyllosphere based on the tree region and the categories of tree height, width, and shade using multivariate analysis of variance (MANOVA) tests.

Pearson’s correlation tests were used to assess the Mantel correlations between the phyllosphere microbiome of the individual *M. grandiflora* trees and the distance (km) between each tree. A permutational analysis of variance (PERMANOVA) was used to assess the differences in the bacterial community composition of the phyllosphere, based on Bray–Curtis dissimilarity scores, between the sampled tree region, and between the categories of tree height, width, and shade. The homogeneity of variance between the groups was analyzed using *permutest* and *betadisper* in “vegan” [38]. The *pairwise.adonis* function from the “pairwiseAdonis” package [39] was used for post hoc analyses. Non-metric multidimensional scaling plots (NMDS) were used to visualize the Bray–Curtis dissimilarity scores between the different trees. The ASVs that were found at >1% relative abundance of all the ASVs and were present in >75% of all the samples and were considered the “core” microbiome of *M. grandiflora*, as determined by the “microbiome” package [40]. To assess the spatial autocorrelation of the bacterial community composition, a distance-based Moran’s eigenvector mapping (dbMEM) analysis was performed using the “adespatial” package [41]. This analysis identifies the spatially structured variation in community data and calculates significant spatial eigenvectors, which were subsequently included as predictors in the PERMANOVA model to examine their contribution to variations in phyllosphere microbiome composition. Significant eigenvectors were selected based on their association with a Bray–Curtis dissimilarity matrix and included in the final model as spatial covariates.

## 3. Results

After the removal of singletons, a total of 6629 ASVs remained in the dataset, with 112,044 ± 13,240 mean sequences (±standard error) recovered per sample. The 87 samples retained for phyllosphere community analyses had a mean Good’s coverage score of 94.8%.

**Figure 2:**
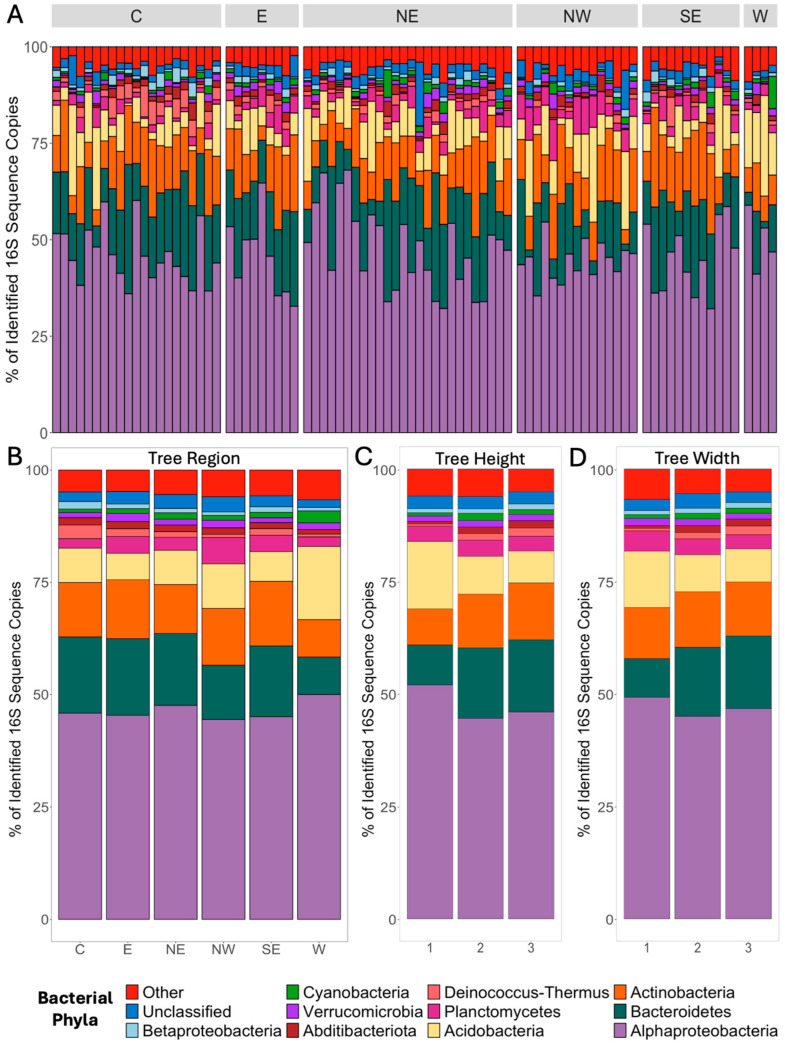
Proportions of the most abundant bacterial phyla in the phyllosphere of 87 *Magnolia grandiflora* trees sampled across a 60 km^2^ area in a small town (Oxford, MS, USA). Bacterial communities were characterized by sequencing the 16S rRNA gene. “Unclassified” represents the bacterial sequences that were not identified to a phylum, while “Other” represents the bacterial sequences identified to a phylum but not classified within the most abundant phyla. The proportion of the major bacterial phyla was determined for each individual tree grouped by each of the six sampling regions (**A**), and subsequently grouped based on the average proportion of each phylum within each region (**B**), and three categories of increasing tree height (**C**) and width (**D**).

The most common bacterial phylum/subphylum recovered from the *M. grandiflora* phyllosphere were Alphaproteobacteria (46.2% of all sequences), Bacteroidetes (15.3%), Actinobacteria (12.1%), Acidobacteria (8.1%), Planctomycetes (3.4%), Deinococcus-Thermus (1.6%), Abditibacteriota (1.5%), Verrucomicrobia (1.4%), Cyanobacteria (1.2%), and Betaproteobacteria (1.2%; Figure 2A). Another 23 phyla each accounted for <1% of the dataset (4.4% in total) and 2.7% of the sequences were identified as bacteria but could not be classified further (i.e., Unclassified). The proportions of Acidobacteria, Deinococcus-Thermus, Planctomycetes, and Betaproteobacteria in the phyllosphere were significantly different between the tree regions (MANOVA; F = 2.44 –8.41, *p* < 0.001–0.05; Figure 2B).

The assigned tree height and width categories were correlated (r = 0.486, *p* < 0.001). In terms of height categories, the proportions of Bacteroidetes (F = 4.59, *p* < 0.05), Actinobacteria (F = 5.21, *p* < 0.05), Deinococcus–Thermus(F = 4.59, *p* < 0.05), and Abditibacteriota (F = 7.69, *p* < 0.01) were significantly greater in the tall (height category 3) and medium (height category 2) trees compared to the short (height category 1) trees, whereas the proportion of Acidobacteria was significantly greater in the short trees (F = 11.6, *p* < 0.01; Figure 2C). The proportions of Deinococcus–Thermusin the phyllosphere were also significantly greater in the wider trees (width categories 2 and 3) compared to the thinner trees (width category 1; F = 4.95, *p* < 0.05; Figure 2D). The trees that were categorized as the most shaded (shade category 3) had significantly lower proportions of Abditibacteriota (F = 3.99, *p* < 0.05) in their phyllosphere compared to those that were less shaded (shade category 1 and 2).

At a finer taxonomic level, the most commonly detected bacterial families in the *M. grandiflora* phyllosphere were Methylobacteriaceae (10.7% of all sequences), Sphingomonadaceae (8.8%), Hymenobacteraceae (8.6%), Microbacteriaceae (6.0%), Acetobacteraceae (4.9%), Cytophagaceae (4.6%), Isosphaeraceae (3.1%), Pseudonocardiaceae (2.9%), Deinococcaceae (1.6%), Abditibacteriaceae (1.5%), Methylocystaceae (1.4%), Beijerinckiaceae (1.3%), and Sphingobacteriaceae (1.0%) with 33.1% of the sequences not identified to the family level. The “core” microbiome of the *M. grandiflora* phyllosphere (i.e., the most abundant ASVs present in >75% of samples) was identified as comprising ASV 2 (in 98.9% or 86 of 87 samples; classified as *Methylobacterium phyllosphaerae*), ASV 1 (97.8% of samples; Rhizobiales), ASV 5 (86.2% of samples; *Amnibacterium*), ASV3 (85.1% of samples; *Methylobacterium aerolatum*), and ASV4 (78.2% of samples; Rhizobiales). The relative abundance of ASV2 (*Methylobacterium phyllosphaerae*) and ASV4 (Rhizobiales) in the phyllosphere were significantly different based on the assigned category of tree height (MANOVA; F = 3.63–10.33, *p* < 0.001–0.05). Shorter trees (height category 1) had a significantly higher relative abundance of ASV2 (15.3% relative abundance) compared to the tallest trees (height category 3; 10.6%), and of ASV4 (21.6%) compared to both the medium (height category 2; 0.98%) and tall trees (1.07%).

**Figure 3:**
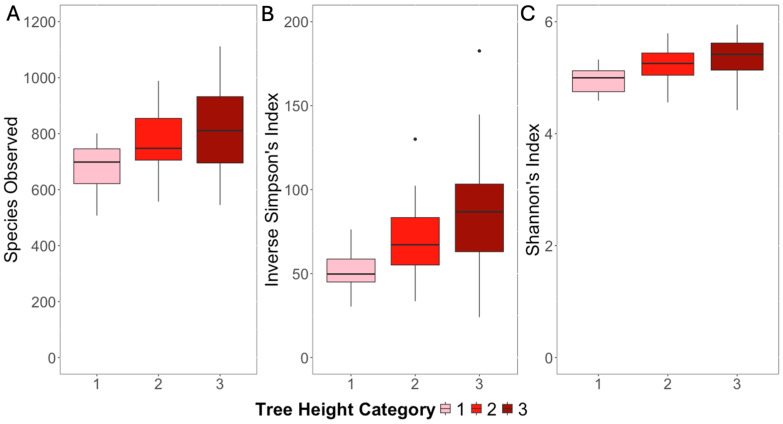
The relationship between the alpha diversity metrics (species observed (**A**); Inverse Simpson’s Index (**B**); and Shannon’s Index (**C**)) of the bacterial phyllosphere communities and the categories of the tree height of 87 *Magnolia grandiflora* trees. The tree height was categorized from 1 (short) to 3 (tall). All the measured alpha diversity metrics were significantly greater in the medium (category 2) and tall (category 3) trees (F = 3.38−4.22, *p* < 0.05) compared to the shortest trees (category 1).

The bacterial species richness (species observed) of the phyllosphere bacterial communities ranged from 507 to 1255, while the diversity (Inverse Simpson’s Index) and evenness (Shannon’s Index) ranged from 24.0 to 182.6 and 4.42–6.19, respectively. All three of these alpha diversity indices were significantly related to the assigned categories of tree height (F = 3.38–4.22, *p* < 0.05; Figure 3A–C), and all three metrics of alpha diversity were significantly greater in the medium and tall trees (height categories 2 and 3) compared to the shorter trees (height category 1; *p*-adj < 0.001–0.05). None of the three indices were significantly related to the categories of tree width, degree of shade, or degree of development (roads, buildings) (*p* > 0.05). The Inverse Simpson’s Index was significantly different in the phyllosphere bacterial communities based on the region of sampling (F = 3.05, *p* < 0.05), with the trees sampled in the east region having significantly higher bacterial species diversity than those in the southeast and west regions (*p*-adj < 0.05). There were no significant relationships between the distance of the trees to nearby roads and the alpha diversity metrics (*p* > 0.05).

**Figure 4:**
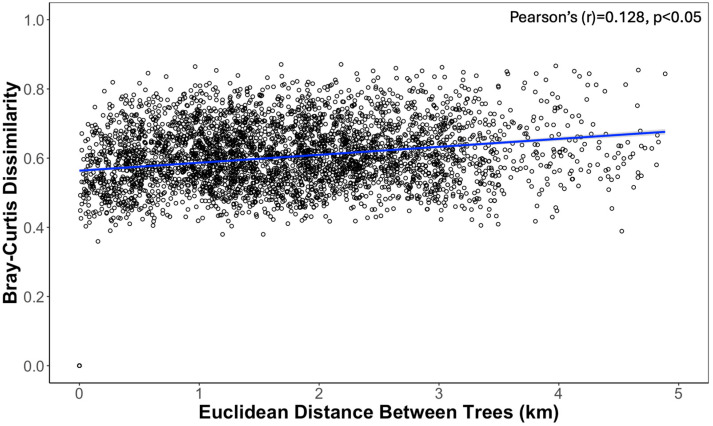
Correlation of spatial distance and microbiome dissimilarities between the bacterial phyllosphere of 87 *Magnolia grandiflora* trees, within a 60 km^2^ area, based on Bray–Curtis dissimilarity scores. Correlations were tested using Pearson’s correlation tests, with Mantel’s correlation coefficient (r) and significance level indicated on the plot. The blue regression line represents the overall model with the variation (standard error) of the model outlined in gray.

**Figure 5:**
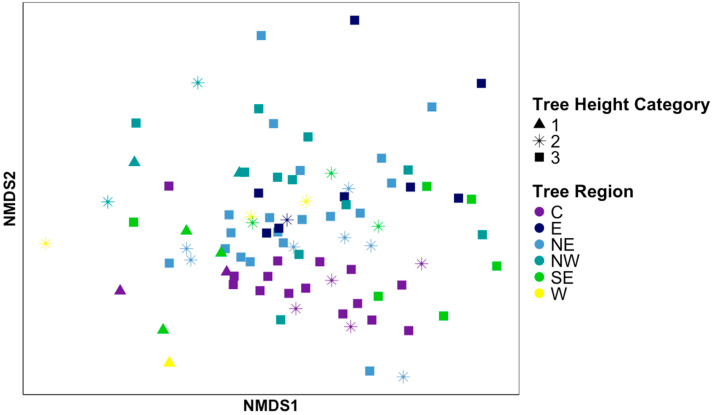
Patterns in bacterial phyllosphere composition of 87 *Magnolia grandiflora* trees between the six sampling regions of a 60 km^2^ area and tree height categories based on Bray–Curtis dissimilarity scores. Each point represents the bacterial community from the phyllosphere of one *M. grandiflora* tree, with the colors separating the tree sampling regions and the shapes differentiating the tree height categories (short = 1, medium = 2, tall = 3). Bacterial community composition of the *M. grandiflora* phyllosphere was significantly different between the sampling region (PERMANOVA; R^2^ = 0.114, F = 2.38) and the tree height (R^2^ = 0.05, F = 2.42, *p* < 0.001).

The composition of the bacterial communities in the phyllosphere, as determined by Bray–Curtis dissimilarity scores, was weakly but significantly correlated with the absolute distance between the trees (Mantel; Pearson’s (r) = 0.128, *p* < 0.05; Figure 4). Additionally, there were significant differences in the *M. grandiflora* phyllosphere between the regions of the town sampled (PERMANOVA; R^2^ = 0.114, F = 2.38, *p* < 0.001; Figure 5), which was driven by the differences between the trees sampled in the central region compared to the trees in the northwest (R^2^ = 0.105, F = 3.98, *p*-adj < 0.05), east (R^2^ = 0.100, F = 3.05, *p*-adj < 0.05), and northeast (R^2^ = 0.058, F = 2.79, *p*-adj < 0.05) regions. The distance of the trees to a road was not significantly related to the bacterial community composition of the phyllosphere (*p* > 0.05). Significant spatially autocorrelated eigenvectors (5 of 18 MEM eigenvectors) from the dbMEM analysis were added to the PERMANOVA model. The regional effects remained significant (R^2^ = 0.11, F = 2.17, *p* < 0.001), with several dbMEM eigenvectors also contributing to community variation. Specifically, MEM5, MEM13, and MEM16 were significantly associated with the bacterial community composition (R^2^ = 0.023, 0.018, 0.019, respectively, *p* < 0.01–0.05) of the phyllosphere. The phyllosphere bacterial community composition was associated with the assigned category of tree height (R^2^ = 0.05, F = 2.42, *p* < 0.001; Figure 5), with a significant interaction effect between the tree height and the region of trees (R^2^ = 0.06, F = 1.30, *p* < 0.05). There was no significant difference in the phyllosphere microbiomes based on the categories of tree width or shade, or the category of site development (*p* > 0.05 for all). Pairwise comparisons of the tree height revealed that the shorter trees (height category 1) had significantly different bacterial communities from those of the medium (height category 2; R^2^ = 0.10, F = 2.74, *p*-adj < 0.01) and tall trees (height category 3; R^2^ = 0.06, F = 3.89, *p*-adj < 0.01). Beta-dispersion was not detected amongst the groups with significant differences in their bacterial community composition (i.e., tree height category or region sampled; permutest ANOVA; *p* > 0.05).

## 4. Discussion

In this study, the phyllosphere bacterial community of 87 Southern magnolia (*Magnolia grandiflora*) trees, sampled across a 60 km^2^ area, was characterized to investigate the local scale biogeographic patterns in these microbial communities. A prior study characterized the bacterial communities on *M. grandiflora* over a much smaller area (<0.5 km^2^) in the same region, attributing the patterns in diversity and community structure to small-scale environmental factors and the tree sampling location [26]. Our study aimed to broaden the understanding of intermediate/local scale geographic spatial patterns, primarily relating phyllosphere bacterial community composition and species diversity to the location of each tree relative to others. Similarly to the findings of the prior study, our study demonstrated a weak correlation between the spatial distance and bacterial community composition (as shown from both Mantel and dbMEM analyses), suggesting that geographic proximity impacts community assembly at intermediate or local scales. Significantly correlated eigenvectors (dbMEM) suggested that the spatial structure at different scales plays a role in shaping the bacterial communities of the *M. grandiflora* phyllosphere. In addition to looking at the effects of the distance between the sampled trees on the phyllosphere community composition, we made categorical estimates of the tree properties such as the height, weight, shading, and site development to allow for an initial examination of their role in influencing the magnolia phyllosphere community. Such categorical assessments are somewhat crude but were necessary given our short timeline of sampling all the trees in a single day. Of these categorical estimates, only the height category of *M. grandiflora* trees, as determined by binning into one of three categories, played a significant role in determining the bacterial community composition and community alpha diversity. These results align with the idea that spatial distance, local environmental conditions, and host characteristics can all influence the microbial community composition of the phyllosphere, as demonstrated in other studies [7,15,25,26].

In addition to the greater spatial distance between the trees relating to phyllosphere community dissimilarity, the bacterial phyla found on the trees sampled in our study resembled those in the prior study [26]. Both of the studies identified Proteobacteria as the most prominent phylum, with Alphaproteobacteria as the most prevalent subphylum, followed by high proportions of Bacteroidetes, Actinobacteria, and Acidobacteria. The two studies occurred in the same region, so similarities are not surprising, but this also suggests that members of these bacterial phyla are common constituents of the *M. grandiflora* phyllosphere in this region. While they were separated by nine years, the prior study occurred at a similar time of year (February) to the current study (March). Those months were chosen to minimize any confounding influences of the surrounding deciduous trees (which have yet to develop leaves by that time of year in this region) on the evergreen *M. grandiflora* phyllosphere. There is certainly the possibility that seasonal patterns in the phyllosphere could occur [42], with potential influences of climatic conditions such as rainfall, humidity, and temperature that vary over an annual cycle. That said, other studies report the similar dominance of Alphaproteobacteria, Bacteroidetes, Acidobacteria, and Actinobacteria in the phyllosphere communities of other tree species, sampled in other parts of the world [17,24,25,27,43,44], suggesting a broad consistency in the dominant bacterial phyla found in the phyllosphere of trees, regardless of the tree species and/or spatial and temporal variability.

While there was general consistency in the phyla present in the phyllosphere of *M. grandiflora*, there were variations from tree to tree in the proportion of each phylum in the overall community, some of which appeared to be dependent on the region of the area sampled. Acidobacteria tended to account for a larger proportion of the bacterial community of the westernmost sampled trees (i.e., west region), where they accounted for 16.4% of the sequences from the trees in that area, despite accounting for just 8.1% of the sequences when all the trees were considered. In addition to variation in the relative abundance of certain phyla based on where the tree was located, we found that, at this scale (a 60 km^2^ area), the differences in the phyllosphere microbiome between the trees were dependent on the distance between those trees. As stated above, this agrees with the patterns that our group has reported at finer (<0.5 km^2^) spatial scales [26], and also aligns with the findings of a study at much larger spatial scales (over distances of 65–333 km) which found a significant influence of the geographic location on the phyllosphere community of 29 trees [25]. The transfer of the microorganisms between the trees is presumably more likely over shorter distances, potentially explaining the positive relationship of distance–dissimilarity reported by Stone and Jackson [26], and at much larger distances, there is likely to be environmental, even climactic, variation that could explain the broader patterns reported by Noble et al. [25]. The intermediate or local scale sampled in our study is likely related to both hypotheses; the trees further apart were more likely to have dissimilar bacterial communities based on less transfer of bacterial populations between the individual trees further apart, yet covered too small of an area for persistent climactic variation to explain the development of different phyllosphere communities. Importantly, this suggests that the factors influencing the composition and diversity of tree phyllosphere communities are likely different and even dependent on the geographic scale sampled.

The assigned height category of the *M. grandiflora* trees also influenced the phyllosphere structure and diversity. While the low R^2^ value of 0.05 suggests that tree height does not explain a large percentage of the variation in the phyllosphere community structure, this finding was significant and complements that of a previous study where differences in tree height correlated with bacterial community dissimilarity [25]. The taller trees also had significantly greater bacterial species richness, diversity, and evenness compared to the trees of lower heights. These relationships to tree height were apparent even with our assignment of height to one of three categories, an approach that was used because the location of many of the trees sampled, coupled with our approach of sampling all the trees within one day, prohibiting the measurements of the actual tree height. It is possible that stronger correlations between the phyllosphere and host tree properties would be found with more accurate measurements of the height, width, and shading, but those measurements were not made in this study. While local environmental conditions influence tree growth, height is most likely a good indicator of tree age; thus, the relationships between the phyllosphere community characteristics and the height category are also likely to be reflective of the relationships with tree age. As trees age, they accumulate nutrients, fostering more favorable conditions for a diverse array of microbial species [45]. Our diversity–height relationships suggest that older trees may harbor more diverse bacterial phyllosphere communities than younger trees, which is further supported by the observation that the phyllosphere of shorter trees was composed of significantly higher proportions of specific bacterial sequences (ASV2 (*Methylobacterium phyllosphaerae*) and ASV4 (Rhizobiales)). While our study sampled leaves at a consistent height, regardless of the size of the tree, previous studies [7,46] have sampled leaves on tree species at various canopy positions and revealed that the microbiome of leaves at higher positions on trees had less bacterial species richness and diversity than the leaves collected lower in the canopy or closer to the tree trunk. Increased tree height can potentially result in greater sunlight exposure (i.e., UV radiation) and/or greater exposure to rain events, leading to a harsher environment on the leaf surface. Taller trees, particularly those with broader canopies, may also experience greater wind dispersal of bacteria, which could influence the bacterial community composition by facilitating the spread of airborne microbes. Together, these findings highlight the complexity of phyllosphere microbiomes and the variability in bacterial community composition, even within individual trees.

Overall, our findings show that the phyllosphere of *M. grandiflora* is dominated by bacterial phyla such as Alphaproteobacteria, Bacteroidetes, Acidobacteria, and Actinobacteria, although the proportions of these phyla in the community varies between the individual trees. In agreement with our initial hypothesis, our study found that over an area of 60 km^2^ the phyllosphere communities of *M. grandiflora* did show a relationship that was dependent on the distances between the trees, a finding that aligns with studies at finer and broader biogeographic scales and suggests that the spatial scales sampled have a large influence on the phyllosphere microbiome dynamics. The general location of the tree (i.e., region) also influenced the phyllosphere community structure, suggesting fine-scale environmental variability, but the tree height category was consistently a more significant influence on the phyllosphere structure and diversity. However, while significant, this height category explained only a small portion of the overall variation in the phyllosphere, such that other factors relating to the host and physical and environmental characteristics of the tree location are likely to be important. These results underscore the importance of considering both biogeography and host characteristics when studying phyllosphere microbial communities.

## Data Availability

The raw sequences along with GPS coordinates for each individual tree are deposited in the NCBI Sequence Reads Archive under BioProject ID PRJNA1176681.
